# Frequency Domain Analysis of Partial-Tensor Rotating Accelerometer Gravity Gradiometer

**DOI:** 10.3390/s21051925

**Published:** 2021-03-09

**Authors:** Xuewu Qian, Liye Zhao, Weiming Liu, Jianqiang Sun

**Affiliations:** 1 School of Instrument Science and Engineering, Southeast University, Nanjing 210096, China; qianxuewu@lyu.edu.cn (X.Q.); 230198292@seu.edu.cn (W.L.); 2 School of Automation and Electrical Engineering, Linyi University, Linyi 276000, China; sunjianqiang@lyu.edu.cn

**Keywords:** frequency domain, gravity gradiometry, rotating accelerometer, output model

## Abstract

The output model of a rotating accelerometer gravity gradiometer (RAGG) established by the inertial dynamics method cannot reflect the change of signal frequency, and calibration sensitivity and self-gradient compensation effect for the RAGG is a very important stage in the development process that cannot be omitted. In this study, a model based on the outputs of accelerometers on the disc of RGAA is established to calculate the gravity gradient corresponding to the distance, through the study of the RAGG output influenced by a surrounding mass in the frequency domain. Taking particle, sphere, and cuboid as examples, the input-output models of gravity gradiometer are established based on the center gradient and four accelerometers, respectively. Simulation results show that, if the scale factors of the four accelerometers on the disk are the same, the output signal of the RAGG only contains (4k+2)ω (ω is the spin frequency of disc for RAGG) harmonic components, and its amplitude is related to the orientation of the surrounding mass. Based on the results of numerical simulation of the three models, if the surrounding mass is close to the RAGG, the input-output models of gravity gradiometer are more accurate based on the four accelerometers. Finally, some advantages and disadvantages of cuboid and sphere are compared and some suggestions related to calibration and self-gradient compensation are given.

## 1. Introduction

Gravity gradiometry is a well-established geophysical technique that is often used in the search for hydrocarbons. The technology measures small differences in the earth’s gravity field associated with changes in subsurface geology. It also plays a key role in inertial navigation, topographic map matching, and geoscience research [1,2,3]. The father of the pratical gravity gradiometer was Baron Lorand von $E\ddot{o}\mathrm{tv}\ddot{o}s$, a Hungarian nobleman and a physicist and engineer, who succeeded in building and deploying a working torsion balance in the late 1890s. His device was used four independent quantities to measure the horizontal derivatives of the vertical component of the gravity acceleration vector, and was widely used in regional mapping for gas and oil in the early 1900s. The physics unit the ‘eotvos’ or Eu (where 1 Eu = 0.1 mGal/km = 10${}^{-9}$ s${}^{-2}$) is now standard for characterising how sensitive different gravity gradiometers are [4,5]. However, the torsion balance was capable of operating on land only, and it was cumbersome, slow in operation and soon was completely abandoned to the favour of compact and fast-operation gravimeters.

Lockheed Martin Corporation (formerly Bell Aerospace) has pioneered practical gravity gradiometry since 50 years ago, and it is the only company to provide commercial moving-base gravity gradiometers, and until recently, broadly offered two types of gravity gradiometer to the exploration industry: the full-tensor gravity gradiometer (FTGG) system, which was deployed in both airborne and marine modes, and the partial-tensor gravity gradiometer (PTGG) system, typically deployed in airborne mode only. The first-generation FTGG system designed in the 1980s by Ernest Metzger of Bell Aerospace during the 1970s and 1980s for the US Navy [6,7], it is sensed by an umbrella configuration of three rotating discs. Each disc, called a gravity gradient instrument unit (GGI), is mounted on a gyro-stabilized platform, and four accelerometers are equally spaced and mounted around the circumference of a rotating disc. It can measure five independent gravity derivatives and can retrieve the whole gravity gradient tensor, the assembly of GGIs is rotated at constant speed about a vertical axis. Brett gives a more detailed account on the workings of the system [8,9]. The PTGG (FALCON${}^{®}$AGG, with 8 Bell model VII-G accelerometers) was jointly developted by Lockheed Martin and BHP Billiton in the late 1990s, with a noise density is 13Eu/$\sqrt{\mathrm{Hz}}$, and it came into service after a two-year flight test [10,11]. Its basic design consists of eletronically matched pairs of accelerometers, and its outputs are differenced to produce a gravity gradient. Recently, the next-generation instrument FALCON plus provides the highest sensitivity and best spatial resolution data, it provides 20 times better spatial resolution (150 m vs. 3000 m) and up to 10 times higher accuracy (0.1 mGal vs. 1.0 mGal) than conventional airborne gravity.

Lockheed Martin Corporation has made several advances with respect to its gravity gradiometer technology since 2010 [12]. A recent significant advance in technology means that the existing generation of gravity gradiometers has been surpassed by Lockheed Martin’s next-generation instrument called the enhanced FTG (eFTG). The eFTG is the world’s most advanced moving-base gravity gradiometer, possessing a noise floor about three times lower than the FTG and providing data with higher bandwidth. The eFTG system combines the best design elements of both gradiometers, essentially comprising three digital partial tensor discs or GGIs mounted in an FTG configuration. This means the eFTG GGIs have eight accelerometers per GGI with a measurement baseline roughly double that of the FTG accelerometer sepration. This also means the eFTG system has a threefold improvement in signal-to-noise ratio (SNR) over the entire bandwidth. The initial performance of the eFTG is 2.5–4 Eu/$\sqrt{\mathrm{Hz}}$, with follow-on improvements expected over time as the system is fielded. At the same time, a digital version of FTGG (dFTG) has been designed, which represents a 41% volume reduction and a 32% weight reduction from earlier analogue versions. The Lockheed Martin’s gravity gradiometry team has developed the next generation of the full-tensor gravity gradiometer (FTG plus), its performance goal is 0.5 Eu/$\sqrt{\mathrm{Hz}}$ over a pass band from 0.1 mHz to 5 Hz, representing a 20-times improvement over FALCON plus [13].

Gravity gradiometry is an important tool for mineral and hydrocarbon exploration. Advances in technology that enable even better spatial resolution, sensitives, accuracy and noise characteristics will make the gravity gradiometry technique even more important [14,15,16,17]. GGI is a high precision measuring instrument, which is extremely sensitive to its operating environment, so the calibration of gravity gradient is needed before airborne gravity gradiometry can be carried out [18,19]. In the process of airborne gravity gradiometry for the full-tensor airborne gravity gradiometer (FTAGG), the attitude of the carrier and the fuel mass will seriously affect the accuracy of gravity gradiometry. In order to improve the accuracy of airborne gravity gradiometry, a self-gradient compensation model has been proposed for FTAGG [20].

However, the methods mentioned above must be based on accurate mathematical model. A good and accurate mathematical model can achieve twice the result with half the effort. Through the previous analysis, it can be concluded that the output model of gravity gradiometer established by the inertial dynamics method cannot reflect the change of signal frequency. In this paper, we set up an accurate input-output mathematical model of RAGG using frequency domain methods. Taking particle, sphere, and cuboid as examples, the input-output models of gravity gradiometer are established respectively. Simulation results show that the output signal of the accelerometer contains higher harmonics components which are the times of the rotating frequency of the disk. The amplitude of the higher harmonic component in the output signal of the gravity gradiometer is related to the consistency of the scale factor of the accelerometer. These conclusions can provide a theoretical basis for the measurement and improvement of the gravity gradiometer.

## 2. Basic Working Principle of RAGG

**Figure 1:**
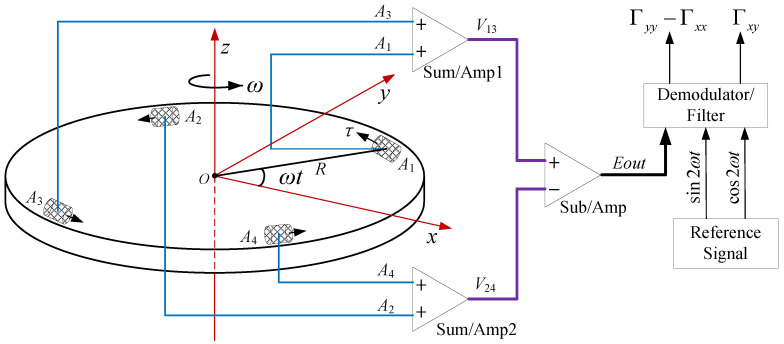
Working principle schematic diagram for the rotating accelerometer gravity gradiometer (RAGG).

Four high precision accelerometers (*A*${}_{1}$ to *A*${}_{4}$) are symmetrically mounted around the circumference of a slow rotating disk. The directions of the sensitive axes of the accelerometers are indicated by a small black arrows, and the sensitive axes of the two adjacent accelerometers are orthogonal to each other, which can eliminate the effect of the host vehicle acceleration. The disc is driven by a high precision motor, which is rotated at constant speed (angular rate ω) about a vertical axis. The working principle schematic diagram for RAGG is shown as Figure 1. We choose the Earth-Centered-Inertial coordinate system and Navigation frame (East-North-Up) coordinate system as the intertial frame (i-frame) and RAGG frame (g-frame), respectively. According to the principle of inertial dynamics, the sensed specific force measured by an accelerometer on the RAGG is:

(1) $$\begin{array}{cc} \; & a^{g}={\ddot{r}}_{ig}^{g}-g_{o}^{g}+2\Omega_{ig}^{g}{\dot{r}}^{g}+\left(\Omega_{ig}^{g}\Omega_{ig}^{g}+\;{\dot{\Omega }}_{ig}^{g}-\Gamma \right)\cdot r^{g} \end{array};$$

where $r_{ig}^{g}$ is the position vector from the origin of the intertial frame to the center of the RAGG, ${\ddot{r}}_{ig}^{g}$ is the kinematic acceleration, $r^{g}$ is the position from the origin of the RAGG center to a accelerometer, $g_{o}^{g}$ is the gravity acceleration vector at the center of the RAGG, $\Omega_{ig}^{g}$ is the skew symmetric matrix of the angular velocity $\omega_{ig}^{g}={\left(\omega_{x},\omega_{y},\omega_{z}\right)}^{T}$ from the g-frame to the i-frame, and the ${\dot{\Omega }}_{ig}^{g}$ is the corresponding angular acceleration, all with coordinates in the g-frame, Γ is the gravitational gradient tensor at the center of the RAGG. For the sake of analysis, assuming that the accelerometers on the disc are rigidly fixed at a specified baseline so that ${\dot{r}}^{g}=0$. The ${\ddot{r}}_{ig}^{g}$, $g_{o}^{g}$ and ${\dot{\omega }}_{ig}^{g}$ are given by

(2) $$\begin{array}{c} \left\{\begin{array}{c} {\ddot{r}}_{ig}^{g}=a_{o}^{g}={\left(a_{ox},a_{oy},a_{oz}\right)}^{T}\\ g_{o}^{g}={\left(g_{ox},g_{oy},g_{oz}\right)}^{T}\\ {\dot{\omega }}_{ig}^{g}={\left({\dot{\omega }}_{x},{\dot{\omega }}_{y},{\dot{\omega }}_{z}\right)}^{T} \end{array}\right. \end{array}.$$

We can calculate the signal of the *j*th accelerometer(*j* = 1,2,3,4) at the direction of the sensitive axis of the corresponding accelerometer by:

(3) $$\begin{array}{cc} \; & a_{j}=K_{I}^{j}{\left[a_{o}^{g}-g_{o}^{g}+\left(\Omega_{ig}^{g}\Omega_{ig}^{g}+{\dot{\Omega }}_{ig}^{g}-\Gamma \right)\cdot r_{j}^{g})\right]}^{T}\cdot \tau_{j} \end{array};$$

where $K_{I}^{j}$ is the scale factor of the *j*th accelerometer, its unit is mA/g (g is the gravity acceleration), $\tau_{j}$ is the uint vector at the direction of the sensitive axis of the *j*th accelerometer $A_{j}$ on the disc. For the RAGG is mounted on a threegimbal stabilized platform, the $\omega_{x}\approx 0,\omega_{y}\approx 0$, so that ${\left(\Omega_{ig}^{g}\Omega_{ig}^{g}\cdot r_{j}^{g}\right)}^{T}\cdot \tau_{j}\approx 0$, the output signal expression of the accelerometer on the rotating disc can be derived [21]. The output signal of the *j*th accelerometer could be expressed as follows:

(4) $$\begin{array}{cc} \; & a_{j}=K_{I}^{j}[\frac{R}{2}\left(\Gamma_{yy}-\Gamma_{xx}\right)\sin2\left(\omega t+\frac{(j-1)\pi }{2}\right)+\dot{\omega }R+R\Gamma_{xy}\cos2\left(\omega t+\frac{(j-1)\pi }{2}\right)+\\ \; & \;\;\;\;\;\;\left(a_{oy}-g_{oy}\right)\cos\left(\omega t+\frac{(j-1)\pi }{2}\right)-\left(a_{ox}-g_{ox}\right)\sin\left(\omega t+\frac{(j-1)\pi }{2}\right)] \end{array};$$

where *R* and $\dot{\omega }$ are the radius, the rotation angular acceleration (${\dot{\omega }}_{z}$) of the disc, respectively. Assuming that the gain of the summing amplifier is 1, then the two summed output signal expressions of the opposing pairs of accelerometers ($A_{1}$ and $A_{3}$, $A_{2}$ and $A_{4}$) are as follows:

(5) $$\begin{array}{c} \left\{\begin{array}{c} V_{13}=\Delta K_{I}^{13}\left[\left(a_{oy}-g_{oy}\right)\cos\omega t-\left(a_{ox}-g_{ox}\right)\sin\omega t\right]+\Sigma K_{I}^{13}R\left[\frac{1}{2}\left(\Gamma_{yy}-\Gamma_{xx}\right)\sin2\omega t+\Gamma_{xy}\cos2\omega t+\dot{\omega }\right]\\ V_{24}=-\Delta K_{I}^{24}\left[\left(g_{oy}-a_{oy}\right)\sin\omega t+\left(a_{ox}-g_{ox}\right)\cos\omega t\right]-\Sigma K_{I}^{24}R\left[\frac{1}{2}\left(\Gamma_{yy}-\Gamma_{xx}\right)\sin2\omega t+\Gamma_{xy}\cos2\omega t-\dot{\omega }\right] \end{array}\right. \end{array};$$

where $\Delta K_{I}^{mn}$ and $\Sigma K_{I}^{mn}$, respectively, demote $K_{I}^{m}-K_{I}^{n}$ and $K_{I}^{m}+K_{I}^{n}$. Assuming the gain of the subtraction amplifier is 1, then the output signal expression of the RAGG is as follows:

(6) $$\begin{array}{cc} E_{out} & =V_{13}-V_{24}\\ \; & =\left[\Delta K_{I}^{13}\left(a_{oy}-g_{oy}\right)+\Delta K_{I}^{24}\left(a_{ox}-g_{ox}\right)\right]\cos\omega t-\left[\Delta K_{I}^{13}\left(a_{ox}-g_{ox}\right)+\Delta K_{I}^{24}\left(a_{oy}-g_{oy}\right)\right]\sin\omega t+\\ \; & \;\Sigma K_{I}^{1234}R\left[\frac{1}{2}\left(\Gamma_{yy}-\Gamma_{xx}\right)\sin2\omega t+\Gamma_{xy}\cos2\omega t\right]+\;\left(\Sigma K_{I}^{13}-\Sigma K_{I}^{24}\right)\dot{\omega } \end{array};$$

where $\Sigma K_{I}^{1234}$ denotes $K_{I}^{1}+K_{I}^{2}+K_{I}^{3}+K_{I}^{4}$. From Equation (6), the inline gravity gradient component ($\Gamma_{yy}-\Gamma_{xx}$) and the cross gravity gradient component $\Gamma_{xy}$ are coupled to the accelerometer scale factor ($\Sigma K_{I}^{1234}$). We should note that on the one hand, if $\Sigma K_{I}^{13}\neq \Sigma K_{I}^{24}$, and the angular acceleration $\dot{\omega }$ contains 2ω signal component. On the other hand, if $\Delta K_{I}^{13}\neq 0$ or $\Delta K_{I}^{24}\neq 0$, and the kinematic acceleration $a_{ox}$ or $a_{oy}$ contains fundamental frequency ω then both of the above-mentioned conditions will affect the accuracy of gravity gradient measurement. In order to eliminate the influence of kinematic acceleration and angular acceleration on gravity gradient measurement, the most effective way is to keep the four accelerometer scale factors consistent [22]. Assuming that the four accelerometers have the same scale factor ($K_{I}^{1}=K_{I}^{2}=K_{I}^{3}=K_{I}^{4}=K_{I}$), the output signal expression of the RAGG can be expressed by:

(7) $$\begin{array}{cc} E_{out} & =4K_{I}R\left[\frac{1}{2}\left(\Gamma_{yy}-\Gamma_{xx}\right)\sin2\omega t+\Gamma_{xy}\cos2\omega t]\right] \end{array}.$$

From Equation (7), the inline gravity gradient component ($\Gamma_{yy}-\Gamma_{xx}$) and the cross gravity gradient component $\Gamma_{xy}$ can be modulated at the sin2ωt and cos2ωt indivdually. Equation (7) seems to give the principle of gravity gradient measurement very well, but only the first term of Taylor expansion of the acceleration component is used to calculate the gravity of accelerometer. Thus, Equation (7) is the approximate output expression of RAGG. If the four accelerometer scale factors are inconsistent, does the output signal for RAGG only contain frequency ω and 2ω? Does the output signal for RAGG have an explicit expression? These questions will be answered below.

## 3. Frequency Domain Analysis for RAGG

### 3.1. Output Signal of Accelerometer for RAGG

**Figure 2:**
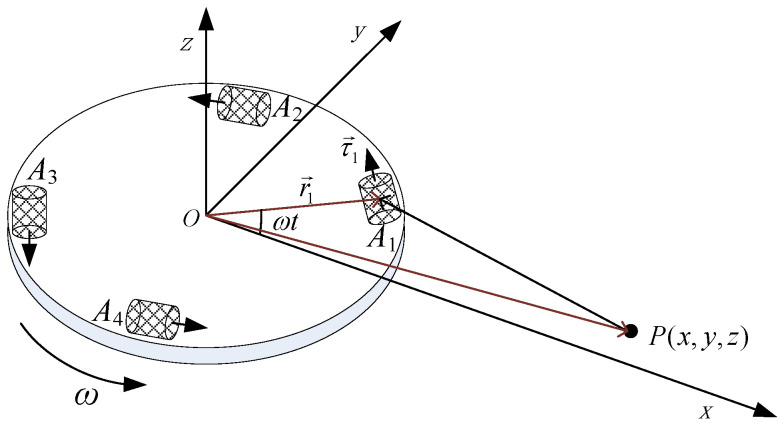
Schematic diagram for the particle act on the RAGG.

In order to facilitate the analysis, it is assumed that there exists a particle *P* with mass *M* outside the RAGG, as shown in Figure 2. According to the law of the universal gravitation, the particle with mass *M* distorts the gravitational field around it, and the change of gravity field will be sensitized by the accelerometer on the RAGG. By combining the output signals of four accelerometers and analyzing the combined signals, the output signal model of the RAGG is obtained.

According to the Newton’s Laws of Motion and the universal gravitation, the output signals at the direction of the sensitive axis of the four accelerometers can be calculated as follows:

(8) $$\begin{array}{cc} a_{j}^{p} & =-\frac{GMK_{I}^{j}}{g{\left|\vec{PA_{j}}\right|}^{3}}\vec{PA_{j}}\cdot \tau_{j} \end{array}.$$

where *G* is the gravitational constant, *M* is the particle mass, g is the gravity acceleration, $\vec{PA_{j}}$ is the position vertor from the particle mass to the accelerometer $A_{j}$. According to vector theory, we get

(9) $$\begin{array}{cc} \vec{PA_{j}} & =\vec{OA_{j}}-\vec{OP}\\ \; & ={\left[\begin{array}{c} R\cos(\omega t+\frac{(j-1)\pi }{2})\\ R\sin(\omega t+\frac{(j-1)\pi }{2})\\ 0 \end{array}\right]}^{T}-{\left[\begin{array}{c} x\\ y\\ z \end{array}\right]}^{T}={\left[\begin{array}{c} R\cos\omega_{j}t-x\\ R\sin\omega_{j}t-y\\ -z \end{array}\right]}^{T} \end{array}.$$

where $\omega_{j}t$ denotes $\omega t+\frac{(j-1)\pi }{2}$, substituting Equation (9) into Equation (8) yields

(10) $$\begin{array}{cc} a_{j}^{p} & =-\frac{GMK_{I}^{j}}{g{\left|\vec{PA_{j}}\right|}^{3}}\vec{PA_{j}}\cdot \tau_{j}=-\frac{GMK_{I}^{j}{\left[\begin{array}{c} R\cos\omega_{j}t-x\\ R\sin\omega_{j}t-y\\ -z \end{array}\right]}^{T}\left[\begin{array}{c} -\sin\omega_{j}t\\ \cos\omega_{j}t\\ 0 \end{array}\right]}{g{\left[R^{2}+x^{2}+y^{2}+z^{2}-2R\left(x\cos\omega_{j}t+y\sin\omega_{j}t\right)\right]}^{3/2}}\\ \; & =-\frac{GMK_{I}^{j}\left(x\sin\omega_{j}t-y\cos\omega_{j}t\right)}{g{\left[R^{2}+x^{2}+y^{2}+z^{2}-2R\left(x\cos\omega_{j}t+y\sin\omega_{j}t\right)\right]}^{3/2}} \end{array}.$$

**Figure 3:**
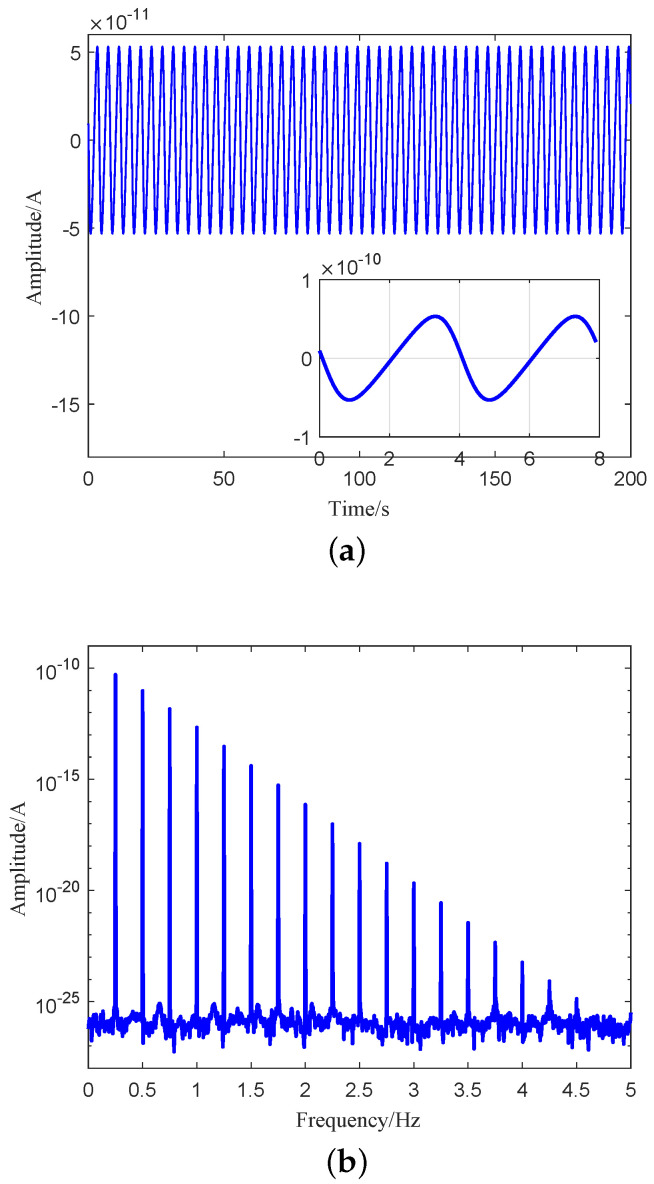
Output signal of accelerometer $A_{1}$. (**a**) Time-domain waveform of accelerometer $A_{1}$. (**b**) Spectrum of accelerometer $A_{1}$.

Assuming that the R=0.1m, ω=0.5π rad/s, M=486kg, $K_{I}^{1}=K_{I}^{2}=K_{I}^{3}=K_{I}^{4}=K_{I}=10\;\mathrm{mA}/g$, the position of the point *P* is (0.8,0.1,0)m, substituting these parameters into Equation (10) yields the output signals of four accelerometers, and the spectrum analysis of the output signal is carried out. Time-domain waveform and spectrum of accelerometer $A_{1}$ are shown in Figure 3a,b, respectively. As shown in Figure 3b, the output signal of accelerometer will include not only fundamental frequency ω, but also the higher-order harmonic components of the spin frequency ω, and the harmonic frequency is an integral multiple of the spin frequency ω, whose amplitude varies in a linear logarithmic.

### 3.2. Output Signal Frequency Domain Expansion of Accelerometer for RAGG

In order to facilitate the analysis, simplifying Equation (10) yields

(11) $$\begin{array}{cc} a_{j}^{p} & =-\frac{GMK_{I}^{j}/g\sqrt{x^{2}+y^{2}}\;\sin\left(\omega_{j}t-\theta \right)}{{\left[R^{2}+x^{2}+y^{2}+z^{2}-2R\sqrt{x^{2}+y^{2}}\;\cos\left(\omega_{j}t-\theta \right)\right]}^{3/2}} \end{array};$$

where θ is the azimuth of the particle. Let

$A_{c}=\frac{GM\sqrt{x^{2}+y^{2}}}{{\left(R^{2}+x^{2}+y^{2}+z^{2}\right)}^{3/2}},\;$ $B_{c}=\frac{2R\sqrt{x^{2}+y^{2}}}{R^{2}+x^{2}+y^{2}+z^{2}}.$

Substituting $A_{c}$ and $B_{c}$ into Equation (11), we get

(12) $$\begin{array}{cc} a_{j}^{p} & =-K_{I}^{j}/gA_{c}\;\sin\left(\omega_{j}t-\theta \right)\left[1-\right.B_{c}\;\cos{\left(\omega_{j}t-\theta \right)}^{-3/2} \end{array}.$$

It should be noted that the detection object is usually outside the gravity gradiometer, so the distance from the particle to the center of RAGG must be larger than the radius of the disc of RAGG, that is, $x^{2}+y^{2}+z^{2}>R^{2}$; therefore, we obtain the following:

(13) $$\begin{array}{c} \frac{2R\sqrt{x^{2}+y^{2}}}{R^{2}+x^{2}+y^{2}+z^{2}}<\frac{\sqrt{x^{2}+y^{2}}}{R}<1 \end{array}.$$

From Equation (13), we get $0<B_{c}<1$, so, used the power series expanding formula to the Equation (12), we obtain the following:

(14) $$\begin{array}{cc} a_{j}^{p} & =-K_{I}^{j}/gA_{c}\;\sin\left(\omega_{j}t-\theta \right)P(t) \end{array};$$

where $P\left(t\right)=1+\sum_{n=1}^{\infty }\frac{2n+1}{2^{2n}}C_{2n}^{n}B^{n}\cos^{n}\left(\omega_{j}t-\theta \right)$, used the power multiplier formula of trigonometric function $\cos^{2n}\left(\omega t\right)=\frac{1}{2^{2n-1}}\left[\sum_{i=0}^{n-1}C_{2n}^{j}B^{n}\cos\left[\left(2n-2j\right)\omega t\right]+\frac{C_{2n}^{n}}{2}\right]$, simplifying Equation (14) yields

(15) $$\begin{array}{cc} a_{j}^{p} & =-K_{I}^{j}/gA_{c}\sum_{k=0}^{\infty }\left\{S_{o}\left(k\right)\sin\left[\left(2k+1\right)\left(\omega_{j}t-\theta \right)\right]+S_{e}\left(k\right)\sin\left[\left(2k+2\right)\left(\omega_{j}t-\theta \right)\right]\right\} \end{array};$$

where $S_{o}\left(k\right)$ and $S_{e}\left(k\right)$ are the odd and even order frequency coefficients, respectively. They are given by

(16) $$\left\{\begin{array}{cc} \; & S_{o}\left(k\right)=\sum_{m=k}^{\infty }\frac{\left(4m+1\right)\left(2k+1\right)C_{4m}^{2m}C_{2m}^{m-k}B_{c}^{2m}}{2^{6m}\left(m+k+1\right)}\\ \; & S_{e}\left(k\right)=\sum_{m=k}^{\infty }\frac{\left(4m+1\right)\left(4m+3\right)\left(k+1\right)C_{4m}^{2m}C_{2m}^{m-k}B_{c}^{2m+1}}{2^{6m+1}\left(m+k+2\right)\left(m+k+1\right)} \end{array};\right.$$

where the *k* = 0,1,2,⋯, the Equation (15) is the output signal frequency domain expansion of accelerometer for RAGG. From Equation (15), we can easily get that the accelerometer output signal contains integer multiple of spin frequency ω. The results are consistent with the frequency distribution of the Figure 3. The value of the frequency coefficients $S_{o}\left(k\right)$ and $S_{e}\left(k\right)$ is directly related to the distance from the particle to the center of the RAGG.

### 3.3. Output Signal Frequency Domain Expression of RAGG

The two summed output signal expressions of the opposing pairs of accelerometers ($A_{1}$ and $A_{3}$, $A_{2}$ and $A_{4}$) are as follows:

(17) $$\begin{array}{c} \left\{\begin{array}{c} V_{13}^{p}=\Delta K_{I}^{13}/gA_{c}\sum_{k=0}^{\infty }S_{o}\left(k\right)\sin\left[\left(2k+1\right)\left(\omega t-\theta \right)\right]-\Sigma K_{I}^{13}/gA_{c}\sum_{k=0}^{\infty }S_{e}\left(k\right)\sin\left[\left(2k+2\right)\left(\omega t-\theta \right)\right]\\ V_{24}^{p}=-\Delta K_{I}^{24}/gA_{c}{\left(-1\right)}^{k}\sum_{k=0}^{\infty }S_{o}\left(k\right)\cos\left[\left(2k+1\right)\left(\omega t-\theta \right)\right]+\Sigma K_{I}^{24}/gA_{c}{\left(-1\right)}^{k}\sum_{k=0}^{\infty }S_{e}\left(k\right)\sin\left[\left(2k+2\right)\left(\omega t-\theta \right)\right] \end{array}\right. \end{array}.$$

We calculate the difference of $V_{13}^{p}$ and $V_{24}^{p}$ by the following:

(18) $$\begin{array}{cc} E_{out}^{p} & =V_{13}^{p}-V_{24}^{p}\\ \; & =\Delta K_{I}^{13}/gA_{c}\sum_{k=0}^{\infty }S_{o}\left(k\right)\sin\left[\left(2k+1\right)\left(\omega t-\theta \right)\right]+\Delta K_{I}^{24}/gA_{c}{\left(-1\right)}^{k}\sum_{k=0}^{\infty }S_{o}\left(k\right)\cos\left[\left(2k+1\right)\left(\omega t-\theta \right)\right]-\\ & \;\;\;A_{c}/g\Sigma K_{Ik}^{1234}\sum_{k=0}^{\infty }S_{e}\left(k\right)\sin\left[\left(2k+2\right)\left(\omega t-\theta \right)\right] \end{array}.$$

where $\Sigma K_{Ik}^{1234}$ demotes $\Sigma K_{I}^{13}+{\left(-1\right)}^{k}\Sigma K_{I}^{24}$, the Equation (18) is the output signal frequency domain expansion of the RAGG. Let k = 0, the output signal of the RAGG will include only the frequency ω and 2ω, then, we get the Equation (18) is simlar to the Equation (6). Amplitude of the odd order frequency coefficients is in inverse proportion to the consistency of the accelerometer scale factors. If $\Delta K_{I}^{13}=0$ and $\Delta K_{I}^{24}=0$, then the odd order harmonic signals can be eliminated, leaving only even harmonic components, and the frequency components of the output signal for the RAGG is (2k+2)ω,(k=0,1,2,⋯). Assuming $a_{m}$ is the general term of frequency coefficient $S_{o}\left(k\right)$, that is, $a_{m}=\frac{\left(4m+1\right)\left(2k+1\right)C_{4m}^{2m}C_{2m}^{m-k}B_{c}^{2m}}{2^{6m}\left(m+k+1\right)}$, and calculating $\lim_{m\to \infty }\frac{a_{m+1}}{a_{m}}$, we get

(19) $$\begin{array}{cc} \; & \lim_{m\to \infty }\frac{a_{m+1}}{a_{m}}=\lim_{m\to \infty }\frac{\frac{\left(4\left(m+1\right)+1\right)\left(2k+1\right)C_{4(m+1)}^{2(m+1)}C_{2(m+1)}^{(m+1)-k}B_{c}^{2(m+1)}}{2^{6(m+1)}\left(\left(m+1\right)+k+1\right)}}{\frac{\left(4m+1\right)\left(2k+1\right)C_{4m}^{2m}C_{2m}^{m-k}B_{c}^{2m}}{2^{6m}\left(m+k+1\right)}}=B_{c}^{2}<1 \end{array}.$$

From Equation (19), based on the theory of the Infinite series convergence, we get the limit of the frequency component $S_{o}\left(k\right)$ exists, which is a constant value. From Equation (18), let *k* is even, and $\Delta K_{I}^{13}=0$, $\Delta K_{I}^{24}=0$, then $\Sigma K_{Ik}^{1234}=4K_{I}$, the frequency components of the output signal for the RAGG is (4k+2)ω, frequency domain expression of the output signal for RAGG is as follows:

(20) $$\begin{array}{cc} E_{out}^{p} & =-4A_{c}K_{I}/g\sum_{k=0}^{\infty }S_{e}\left(k\right)\sin\left[(4k+2)(\omega t-\theta )\right] \end{array}.$$

From Equation (20), the output signal of the RAGG will include only the even-order harmonic components of the spin frequency ω, that is (4k+2)ω, especially when k=0, the magnitude of Equation (20) reflects the magnitude of gravity gradient. The inline gravity gradient component ($\Gamma_{yy}-\Gamma_{xx}$) and the cross gravity gradient component $\Gamma_{xy}$ can be extracted by demodulation, with reference signals of sin2ωt and cos2ωt, respectively.

## 4. Gravity Gradient Signal Model of the RAGG

An accurate gravity gradient signal model is needed to verify the performance index or calibrate the gravity gradient for the RAGG. In this section, the signal models of the RAGG are established by taking particle and cuboid as examples.

### 4.1. Particle as Surrounding Mass

As shown in Figure 2, according to the Newton’s Laws of Motion and the universal gravitation, the expressions of the gravity gradient component of the particle P to the center of the RAGG are as follows:

(21) $$\begin{array}{c} \left\{\begin{array}{cc} \Gamma_{xx}{|}_{\mathrm{PC}} & =\frac{3GMx^{2}}{{\left(x^{2}+y^{2}+z^{2}\right)}^{5/2}}-\frac{GM}{{\left(x^{2}+y^{2}+z^{2}\right)}^{3/2}}\\ \Gamma_{yy}{|}_{\mathrm{PC}} & =\frac{3GMy^{2}}{{\left(x^{2}+y^{2}+z^{2}\right)}^{5/2}}-\frac{GM}{{\left(x^{2}+y^{2}+z^{2}\right)}^{3/2}}\\ \Gamma_{xy}{|}_{\mathrm{PC}} & =\frac{3GMxy}{{\left(x^{2}+y^{2}+z^{2}\right)}^{5/2}} \end{array}\right. \end{array}.$$

For the partial tensor Gravity-Gradiometer, it can only measure gravity gradient information ($\Gamma_{yy}-\Gamma_{xx}$) and $\Gamma_{xy}$, respectively. The combined gravity gradient components can be represented as

(22) $$\begin{array}{c} \left\{\begin{array}{cc} \; & \left(\Gamma_{yy}-\Gamma_{xx}\right){|}_{\mathrm{PC}}=\frac{3GM(y^{2}-x^{2})}{{\left(x^{2}+y^{2}+z^{2}\right)}^{5/2}}\\ \; & \Gamma_{xy}{|}_{\mathrm{PC}}=\frac{3GMxy}{{\left(x^{2}+y^{2}+z^{2}\right)}^{5/2}} \end{array}\right. \end{array}.$$

The Equation (22) is the gravity gradient input-output signal model of the RAGG based on the center of the disc for the particle. According to Equation (20), if k=0, simplifying Equation (20) yields

(23) $$\begin{array}{cc} E_{out}^{p}{|}_{k=0} & =-4A_{c}K_{I}/gS_{e}\left(0\right)\cos2\theta \sin2\omega t+4A_{c}K_{I}/gS_{e}\left(0\right)\sin2\theta \cos2\omega t \end{array}.$$

By demodulating at the sin2ω and cos2ω individually to the Equation (23), gravity-gradient components based on the accelerometer of the RAGG can be calculated as

(24) $$\begin{array}{c} \left\{\begin{array}{cc} \; & \left(\Gamma_{yy}-\Gamma_{xx}\right){|}_{\mathrm{PA}}=-\frac{2A_{c}S_{e}\left(0\right)\cos2\theta }{gR}\\ \; & \Gamma_{xy}{|}_{\mathrm{PA}}=\frac{A_{c}S_{e}\left(0\right)\sin2\theta }{gR} \end{array}\right. \end{array}.$$

where $\left(\Gamma_{yy}-\Gamma_{xx}\right){|}_{\mathrm{PA}}$ and $\Gamma_{xy}{|}_{\mathrm{PA}}$ denote the actual gravity-gradient components $\left(\Gamma_{yy}-\Gamma_{xx}\right)$ and $\Gamma_{xy}$ for the RAGG, respectively. The Equation (24) should be rewritten as

(25) $$\begin{array}{c} \left\{\begin{array}{cc} \; & \left(\Gamma_{yy}-\Gamma_{xx}\right){|}_{\mathrm{PA}}=-GMP_{c}\left[3+4\sum_{m=1}^{\infty }Q_{c}\left(m\right)\right]\cos2\theta \\ \; & \Gamma_{xy}{|}_{\mathrm{PA}}=GMP_{c}\left[\frac{3}{2}+2\sum_{m=1}^{\infty }Q_{c}\left(m\right)\right]\sin2\theta \end{array}.\right. \end{array}$$

where $P_{c}$, $Q_{c}\left(m\right)$ are given:

(26) $$\begin{array}{c} \left\{\begin{array}{cc} \; & P_{c}=-\frac{\left(x^{2}+y^{2}\right)}{{\left(R^{2}+x^{2}+y^{2}+z^{2}\right)}^{5/2}}\\ \; & Q_{c}\left(m\right)=\frac{\left(4m+1\right)\left(4m+3\right)C_{4m}^{2m}C_{2m}^{m}R^{2m}{\left(x^{2}+y^{2}\right)}^{m}}{2^{4m-1}\left(m+2\right)\left(m+1\right){\left(R^{2}+x^{2}+y^{2}+z^{2}\right)}^{2m}} \end{array}\right. \end{array}.$$

The Equation (26) is the gravity gradient signal model of the RAGG based on the accelerometers for the particle. According to the relationship between the azimuth angle of the particle *P* and its coordinates, there exists the following triangular relation

(27) $$\begin{array}{ccc} \sin2\theta & =\frac{2xy}{x^{2}+y^{2}},\;\;\cos2\theta & =\frac{x^{2}-y^{2}}{x^{2}+y^{2}} \end{array}.$$

Substituting Equation (27) into Equation (22), the Equation (22) can be rewritten as

(28) $$\begin{array}{c} \left\{\begin{array}{cc} \; & \left(\Gamma_{yy}-\Gamma_{xx}\right){|}_{\mathrm{PC}}=-3GMF_{c}\cos2\theta \\ \; & \Gamma_{xy}{|}_{\mathrm{PC}}=\frac{3GMF_{c}}{2}\sin2\theta \end{array}\right. \end{array}.$$

where $F_{c}$ is given:

(29) $$\begin{array}{c} F_{c}=\frac{\left(x^{2}+y^{2}\right)}{{\left(x^{2}+y^{2}+z^{2}\right)}^{5/2}} \end{array}.$$

Comparing Equation (25) and Equation (28), when the distance from the particle to the center of the RAGG is much larger than the radius of the RAGG, that is, when $x^{2}+y^{2}+z^{2}\gg R^{2}$ is satisfied, Equation (25) can be written as

(30) $$\begin{array}{c} \left\{\begin{array}{cc} \; & \left(\Gamma_{yy}-\Gamma_{xx}\right){|}_{\mathrm{PA}}\approx -\frac{3GM(x^{2}+y^{2})\cos2\theta }{{\left(R^{2}+x^{2}+y^{2}+z^{2}\right)}^{5/2}}\approx \left(\Gamma_{yy}-\Gamma_{xx}\right){|}_{\mathrm{PC}}\\ & \Gamma_{xy}{|}_{\mathrm{PA}}\approx \frac{3GM(x^{2}+y^{2})\sin2\theta }{2{\left(R^{2}+x^{2}+y^{2}+z^{2}\right)}^{5/2}}\approx \Gamma_{xy}{|}_{\mathrm{PC}} \end{array}\right. \end{array}.$$

**Figure 4:**
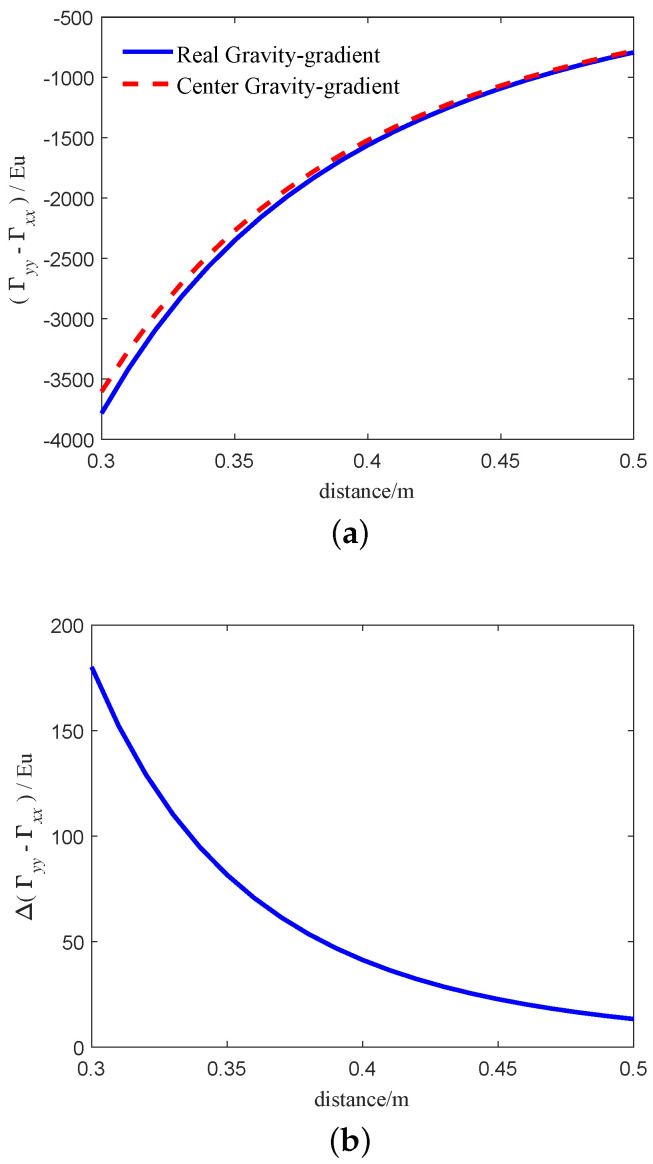
Gravity-gradient of the two models based on the particle. (**a**) The relationship between the calculated results of the two models and the distance. (**b**) The relationship between the calculated results error of the two models and the distance.

From Equation (25), Equation (28), we can get that the gravity gradient calculated by Equation (25) is more accurate than Equation (28) when the center of environmental mass is close to the center of the RAGG. In order to compare the difference between the two models intuitively, we simulate the two models. The simulation parameters refer to Section 3.1. The simulation results are shown in Figure 4. The blue solid line and the red dashed line in Figure 4a indicates the real gravity-gradient of RAGG calculated by Equation (25) and center gravity-gradient calculated by Equation (28), respectively. The results calculated by the two models are not equal, mainly because of the distance from the accelerometer to the center of the RAGG. Figure 4b indicates the calculation error between the two models. From Figure 4b, as the distance between the particle and the center of the GGI decreases, the error between the two models becomes larger and larger. When the distance is 0.3 m, the calculation error between the two models exceeds 100 Eu. Considering the installation of accelerometer, the distance cannot be zero. Moreover, reducing the radius of the GGI will reduce the SNR of gravity gradient signal. For example, the radius of the RAGG used in FTG system by Lockheed Martin Company was 0.1 m in the early stage. Later, the radius of the GGI on FALCON${}^{®}$AGG system of partial tensor airborne gravity gradiometer developed in cooperation with BHP was changed to 0.2 m. After testing, it was found that the noise of FALCON${}^{®}$AGG system was greatly reduced, and the exploration precision of FALCON${}^{®}$AGG system was better than that of FTG system. The selection of disk radius should be based on the actual application. In summary, when the surrounding masses are close to the center of RAGG, Equation (28) will lead to large calculation errors; it cannot accurately describe the input-output relationship of the RAGG. Thus, the gravity gradient calculated by Equation (25) is more accurate.

### 4.2. Cuboid as Surrounding Mass

**Figure 5:**
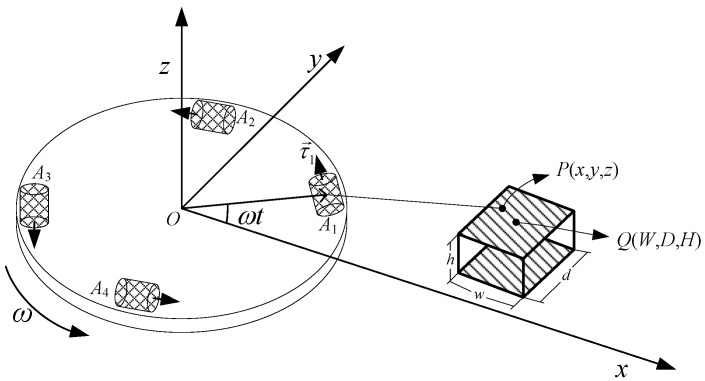
Schematic diagram for the cuboid act on the RAGG.

The width, depth, and height of the cuboid are denoted by *w*, *d* and *h*, respectively, the density of the cuboid is denoted by ρ, the centroid coordinate of the cuboid is Q(W,D,H), and the coordinate of any point in the cuboid is P(x,y,z), as shown in Figure 5.

According to the expressions of the triangular relation of position and azimuth Equation (27), the expressions of the gravity gradient component of the cuboid to the center of the RAGG are as follows:

(31) $$\begin{array}{c} \left\{\begin{array}{cc} \; & \left(\Gamma_{yy}-\Gamma_{xx}\right){|}_{\mathrm{CC}}=-3G\rho \int_{W-\frac{w}{2}}^{W+\frac{w}{2}}dx\int_{D-\frac{d}{2}}^{D+\frac{d}{2}}dy\int_{H-\frac{h}{2}}^{H+\frac{h}{2}}F_{c}\cos2\theta dz\\ \; & \Gamma_{xy}{|}_{\mathrm{CC}}=3G\rho \int_{W-\frac{w}{2}}^{W+\frac{w}{2}}dx\int_{D-\frac{d}{2}}^{D+\frac{d}{2}}dy\int_{H-\frac{h}{2}}^{H+\frac{h}{2}}\frac{F_{c}\sin2\theta }{2}dz \end{array}\right. \end{array}.$$

From the gravity gradient signal model Equation (25) of the RAGG for the particle, it is easy to obtain the result that the gravity gradient component $\Gamma_{yy}-\Gamma_{xx}$ and $\Gamma_{xy}$ under the influence of the cuboid yields:

(32) $$\begin{array}{c} \left\{\begin{array}{cc} \; & \left(\Gamma_{yy}-\Gamma_{xx}\right){|}_{\mathrm{CA}}=-G\rho \int_{W-\frac{w}{2}}^{W+\frac{w}{2}}dx\int_{D-\frac{d}{2}}^{D+\frac{d}{2}}dy\int_{H-\frac{h}{2}}^{H+\frac{h}{2}}P_{c}\left[3+4\sum_{m=1}^{\infty }Q_{c}\left(m\right)\right]\cos2\theta dz\\ \; & \Gamma_{xy}{|}_{\mathrm{CA}}=G\rho \int_{W-\frac{w}{2}}^{W+\frac{w}{2}}dx\int_{D-\frac{d}{2}}^{D+\frac{d}{2}}dy\int_{H-\frac{h}{2}}^{H+\frac{h}{2}}P_{c}\left[\frac{3}{2}+2\sum_{m=1}^{\infty }Q_{c}\left(m\right)\right]\sin2\theta dz \end{array}\right. \end{array}.$$

The Equation (32) is the gravity gradient signal model of the RAGG based on the accelerometers for the cuboid. Comparing Equation (31) and Equation (32), when the distance from the cuboid to the center of the RAGG is much larger than the radius of the RAGG. That is, when $x^{2}+y^{2}+z^{2}\gg R^{2}$ is satisfied, Equation (32) can be written as

(33) $$\begin{array}{c} \left\{\begin{array}{cc} \; & \left(\Gamma_{yy}-\Gamma_{xx}\right){|}_{\mathrm{CA}}\approx -3G\rho \int_{W-\frac{w}{2}}^{W+\frac{w}{2}}dx\int_{D-\frac{d}{2}}^{D+\frac{d}{2}}dy\int_{H-\frac{h}{2}}^{H+\frac{h}{2}}\frac{(x^{2}+y^{2})\cos2\theta }{{\left(R^{2}+x^{2}+y^{2}+z^{2}\right)}^{5/2}}dz\approx \left(\Gamma_{yy}-\Gamma_{xx}\right){|}_{\mathrm{PC}}\\ \; & \Gamma_{xy}{|}_{\mathrm{CA}}=3G\rho \int_{W-\frac{w}{2}}^{W+\frac{w}{2}}dx\int_{D-\frac{d}{2}}^{D+\frac{d}{2}}dy\int_{H-\frac{h}{2}}^{H+\frac{h}{2}}\frac{(x^{2}+y^{2})\sin2\theta }{2{\left(R^{2}+x^{2}+y^{2}+z^{2}\right)}^{5/2}}dz\approx \Gamma_{xy}{|}_{\mathrm{PC}} \end{array}\right. \end{array}.$$

**Figure 6:**
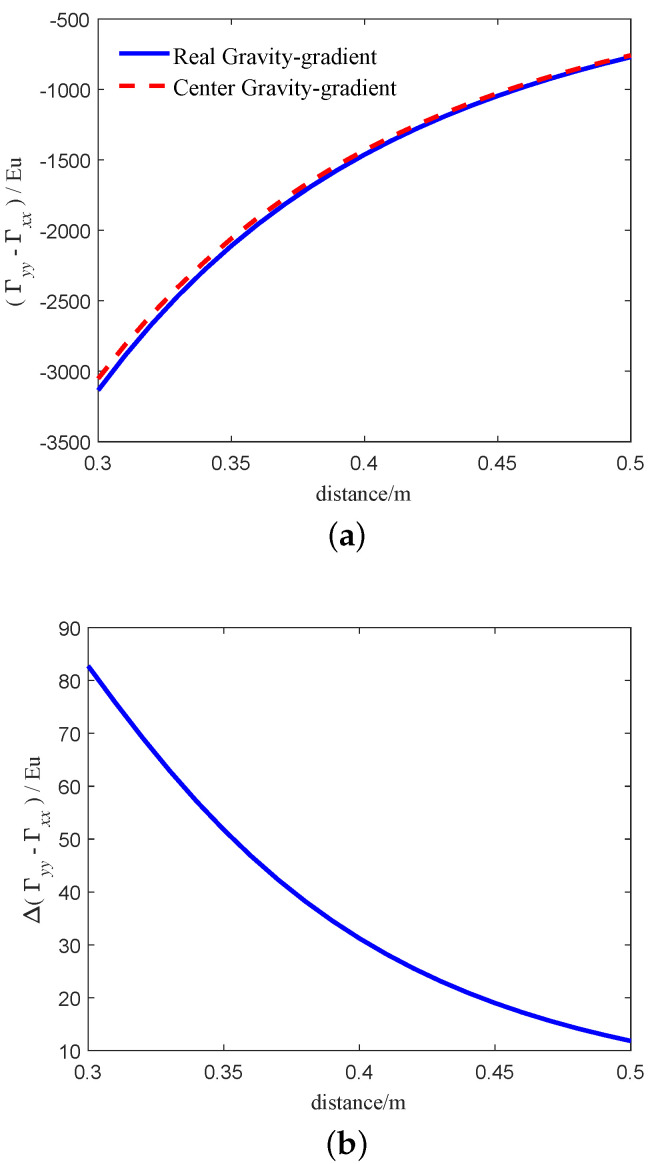
Gravity-gradient of the two models based on the cuboid. (**a**) The relationship between the calculated results of the two models and the distance. (**b**) The relationship between the calculated results error of the two models and the distance.

From Equation (31), Equation (32), we can get that the gravity gradient calculated by Equation (32) is more accurate than Equation (31) when the center of environmental mass is close to the center of the RAGG. In order to compare the difference between the two models intuitively, we simulate the two models. Assuming that *w* = *d* = *h* = 0.3 m, D=0.1m, H=0m, R=0.1m, ω=0.5π rad/s, $\rho =18,000\;\mathrm{kg}/m^{3}$. When the cuboid moves along the *x*-axis, the simulation results of the two models are shown in Figure 6. The blue solid line and the red dashed line in Figure 6a indicates the real gravity-gradient of RAGG calculated by Equation (32) and center gravity-gradient calculated by Equation (31), respectively. The results calculated by the two models are not equal, mainly because of the distance from the accelerometer to the center of the RAGG. Figure 6b indicates the calculation error between the two models. From Figure 6b, as the distance between the cuboid and the center of the RAGG decreases, the error between the two models becomes larger and larger. When the distance is 0.3 m, the calculation error between the two models exceeds 60 Eu.

### 4.3. Sphere as Surrounding Mass

**Figure 7:**
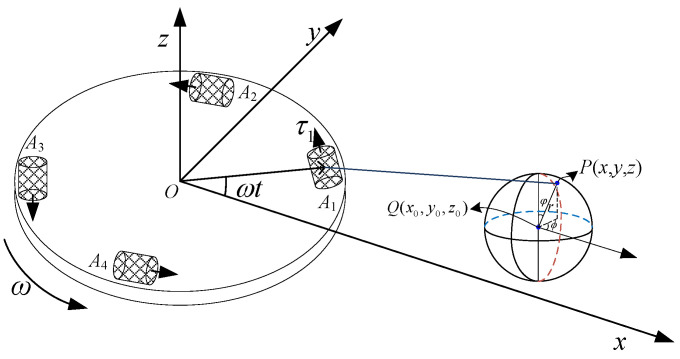
Schematic diagram for the sphere act on the RAGG.

To avoid confusion with the disc radius *R*, assuming that the radius of sphere is $R_{0}$, the density of the cuboid is denoted by ρ, the centroid location of the cuboid is $Q(x_{0},y_{0},z_{0})$, and the location of any point in the cuboid is P(x,y,z), as shown in Figure 7. According to the calculation formula of sphere volume, and the expressions of the triangular relation of position and azimuth Equation (27), the expressions of the gravity gradient component of the sphere to the center of the RAGG are as follows:

(34) $$\begin{array}{c} \left\{\begin{array}{cc} \; & \left(\Gamma_{yy}-\Gamma_{xx}\right){|}_{\mathrm{SC}}=-3G\rho \int_{0}^{R_{0}}r^{2}dr\int_{0}^{\pi }\sin\varphi d\varphi \int_{0}^{2\pi }F_{c}\cos2\theta d\phi \\ \; & \Gamma_{xy}{|}_{\mathrm{SC}}=3G\rho \int_{0}^{R_{0}}r^{2}dr\int_{0}^{\pi }\sin\varphi d\varphi \int_{0}^{2\pi }\frac{F_{c}\sin2\theta }{2}d\phi \end{array}\right. \end{array}.$$

The location for the point P(x,y,z) can be expressed by the spherical coordinate position P(r,φ,ϕ) as follows:

(35) $$\begin{array}{c} \left\{\begin{array}{cc} \; & x=r\sin\varphi \cos\phi +x_{0}\\ \; & y=r\sin\varphi \sin\phi +y_{0}\\ \; & z=r\cos\varphi +z_{0} \end{array}\right. \end{array}.$$

Using the previous method, the gravity gradient component $\Gamma_{yy}-\Gamma_{xx}$ and $\Gamma_{xy}$ under the influence of the sphere can be expressed as:

(36) $$\begin{array}{c} \left\{\begin{array}{cc} \; & \left(\Gamma_{yy}-\Gamma_{xx}\right){|}_{\mathrm{SA}}=-G\rho \int_{0}^{R_{0}}r^{2}dr\int_{0}^{\pi }\sin\varphi d\varphi \int_{0}^{2\pi }P_{c}\left[3+4\sum_{m=1}^{\infty }Q_{c}\left(m\right)\right]\cos2\theta d\phi \\ \; & \Gamma_{xy}{|}_{\mathrm{SA}}=G\rho \int_{0}^{R_{0}}r^{2}dr\int_{0}^{\pi }\sin\varphi d\varphi \int_{0}^{2\pi }P_{c}\left[\frac{3}{2}+2\sum_{m=1}^{\infty }Q_{c}\left(m\right)\right]\sin2\theta d\phi \end{array}\right. \end{array}.$$

The Equation (36) is the gravity gradient signal model of the RAGG based on the accelerometers for the sphere. Comparing Equation (34) and Equation (36), when the distance from the sphere to the center of the RAGG is much larger than the radius of the RAGG, that is, when $x^{2}+y^{2}+z^{2}\gg R^{2}$ is satisfied, Equation (36) can be written as

(37) $$\begin{array}{c} \left\{\begin{array}{cc} \; & \left(\Gamma_{yy}-\Gamma_{xx}\right){|}_{\mathrm{SA}}\approx \left(\Gamma_{yy}-\Gamma_{xx}\right){|}_{\mathrm{SC}}\approx \left(\Gamma_{yy}-\Gamma_{xx}\right){|}_{\mathrm{PC}}\\ \; & \Gamma_{xy}{|}_{\mathrm{SA}}\approx \Gamma_{xy}{|}_{\mathrm{SC}}\approx \Gamma_{xy}{|}_{\mathrm{PC}} \end{array}\right. \end{array}.$$

**Figure 8:**
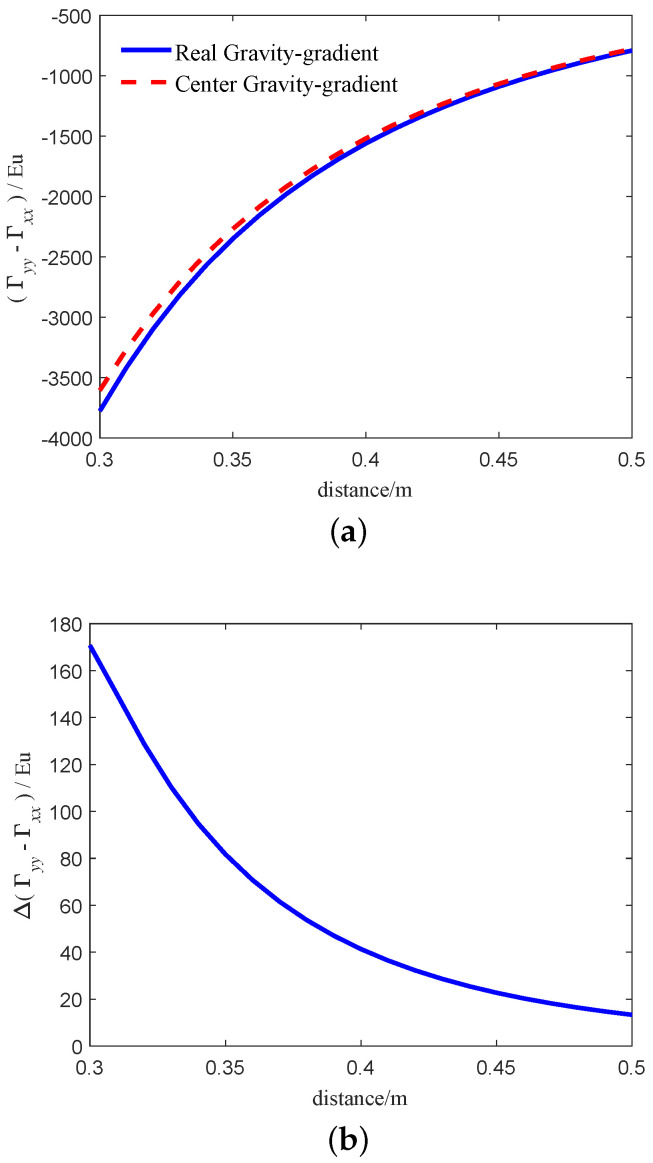
Gravity-gradient of the two models based on the sphere. (**a**) The relationship between the calculated results of the two models and the distance. (**b**) The relationship between the calculated results error of the two models and the distance.

From Equation (34), Equation (36), we can get that the gravity gradient calculated by Equation (36) is more accurate than Equation (34) when the center of environmental mass is close to the center of the RAGG. In order to compare the difference between the two models intuitively, we simulate the two models. Assuming that $y_{0}=0.1\;m$, $z_{0}=0\;m$, R=0.1m, ω=0.5π rad/s, $\rho =18,000\;\mathrm{kg}/m^{3}$, M=486kg (the wight of the sphere), $R_{0}=\sqrt[{3}]{3M/(4\rho \pi )}$. When the sphere moves along the *x*-axis, the simulation results of the two models are shown in Figure 8. The blue solid line and the red dashed line in Figure 8a indicates the real gravity-gradient of RAGG calculated by Equation (36) and center gravity-gradient calculated by Equation (34), respectively. The results calculated by the two models are not equal, mainly because of the distance from the accelerometer to the center of the RAGG. Figure 8b indicates that the calculation error between the two models. From Figure 8b, as the distance between the sphere and the center of the RAGG decreases, the error between the two models becomes larger and larger. When the distance is close to 0.3 m, the calculation error between the two models exceeds 60 Eu.

**Figure 9:**
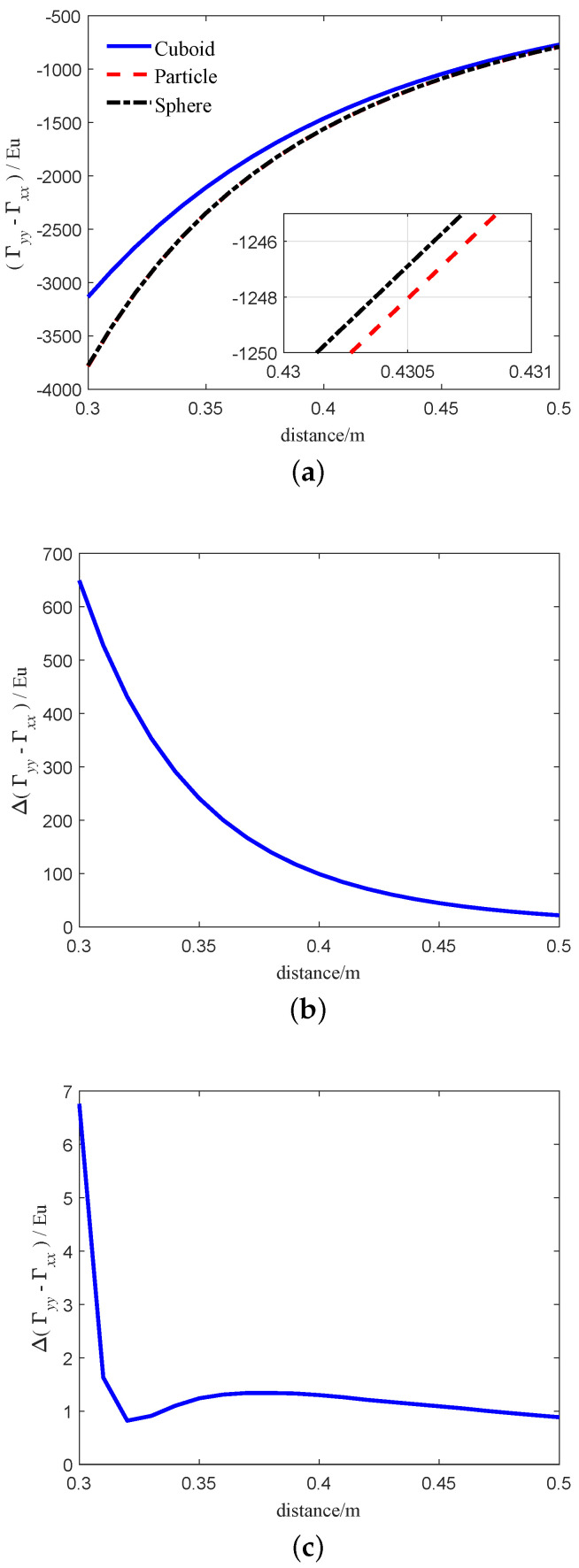
Real-gravity gradient for the RAGG caused by the particle and the cuboid which are the same mass. (**a**) The relationship between the real-gravity gradient for the RAGG caused by the particle and the cuboid and the distance. (**b**) The relationship between the Real-gravity gradient error for the RAGG caused by the particle and the cuboid and the distance. (**c**) The relationship between the Real-gravity gradient error for the RAGG caused by the particle and the sphere and the distance.

Finally, we examine the real-gravity gradient for the RAGG caused by the particle, cuboid, and sphere which are the same mass. The simulation parameters of the models (Equations (25), (32) and (36) of the particle, cuboid and sphere can be refer to the Section 3.1, Section 4.2 and Section 4.3, respectively. The simulation results of the three models are shown in Figure 9. The blue solid line, red dashed line and black dash-dotted line in Figure 9a indicates the real gravity-gradient of RAGG calculated by Equation (32) based on the cuboid, Equation (25) based on the particle and Equation (36) based on the sphere, respectively. The results calculated by the three models are not equal, mainly because of the shape of the surrounding masses. Figure 9b indicates that the calculation error between the cuboid and the particle. From Figure 9b, as the distance between the surrounding masses and the center of the RAGG decreases, the error between the two models becomes larger and larger. When the distance is 0.3 m, the calculation error between the two models exceeds 100 Eu. The model’s calculation error between the sphere and the particle is smaller than that between the cuboid and the particle. Figure 9c indicates that the calculation error between the sphere and the particle. From Figure 9c, as the distance between the surrounding masses and the center of the RAGG decreases, the error between the two models does not change much. When the distance is greater than 0.32 m, the calculation error between the two models is within 1 Eu. From the above analysis, it can be concluded that the calculation accuracy of the model is related to the shape of the detected object. Compared with the cuboid, the calculation error caused by the sphere is smaller. In addition, under the same quality, the larger the volume, the greater the calculation error, the stronger the nonlinearity between the center of the detected object and the gravity gradient.

## 5. Conclusions

The output signal frequency domain expression of the single accelerometer and the summed output signal domain expression of the opposing pairs of accelerometers were analyzed, the laws and the frequency domatin components of the total output signal for the RAGG were studied, and the output signal of the RAGG included only the even-order harmonic components (4k+2)ω. Prior to the gravity gradiometer testing or commercial use, gravity gradient instrument calibration is required, and accurate input and output models are required for calibration. Therefore, in this paper, in order to facilitate future engineering tests, we present a gravity gradient input and output model based on the particle and cuboid for the RAGG, respectively. The simulation results show that the error between the two models gradually increases with the decrease of the distance from the surrounding masses to the center of the RAGG. At the same distance, the error between the two models is related to the shape of the surrounding masses, material, and other parameters. When the mass of the surrounding masses are the same, the smaller the distance from the center of the RAGG, the larger the gradient error caused. At this time, the influence of the radius of the RAGG cannot be ignored. Therefore, when the surrounding masses are close to GGI, the input-output model of the RAGG center is inaccurate.

Because the cuboid is easy to process and place, the cuboid is often used as a gravity gradient effect detection device. In the gravity gradient effect test or gravity gradient calibration experiment, first of all, it is necessary to select the appropriate shape of the object to be detected, and then choose the appropriate size and material according to the output range and calibration accuracy of the gradiometer. Finally, the relationship between the distance and the output of a specific detection object can be accurately calculated by the input-output model of the RAGG based on the accelerometers.

## Data Availability

Not applicable.
